# A fast and efficient path elimination algorithm for large-scale multiple common longest sequence problems

**DOI:** 10.1186/s12859-022-04906-5

**Published:** 2022-09-07

**Authors:** Changyong Yu, Pengxi Lin, Yuhai Zhao, Tianmei Ren, Guoren Wang

**Affiliations:** 1 College of Computer Science and Engineering, Northeastern University, Shenyang, China; 2 School of Computer Science and Technology, Beijing Institute of Technology, Beijing, China

**Keywords:** Multiple longest common subsequences (MLCS), The branch and bound, Mini-MLCS

## Abstract

**Background:**

In various fields, searching for the Longest Common Subsequences (LCS) of Multiple (i.e., three or more) sequences (MLCS) is a classic but difficult problem to solve. The primary bottleneck in this problem is that present state-of-the-art algorithms require the construction of a huge graph (called a direct acyclic graph, or DAG), which the computer usually has not enough space to handle. Because of their massive time and space consumption, present algorithms are inapplicable to issues with lengthy and large-scale sequences.

**Results:**

A mini Directed Acyclic Graph (mini-DAG) model and a novel Path Elimination Algorithm are proposed to address large-scale MLCS issues efficiently. In mini-DAG, we employ the branch and bound approach to reduce paths during DAG construction, resulting in a very mini DAG (mini-DAG), which saves memory space and search time.

**Conclusion:**

Empirical experiments have been performed on a standard benchmark set of DNA sequences. The experimental results show that our model outperforms the leading algorithms, especially for large-scale MLCS problems.

## Introduction

In various fields such as cancer treatment [1], cancer detection [2], protein sequence classifying [3], gene data searching [4], and gene data analyzing [5], searching for the Longest Common Subsequences (LCS) of Multiple (i.e., three or more) sequences (MLCS) is a classic but difficult problem to solve. With the increase in the number of sequences and the advancement of biotechnology, this problem is usually divided into two categories. The first is to find the longest common subsequence between two sequences, called the LCS problem; the second is to find the longest common subsequence among three or more sequences, called the MLCS problem.

In the past few decades, many algorithms dedicated to solving LCS problems have been proposed; for example, Sankoff [6] published a paper in which he described how to use the dynamic programming (DP) algorithm to determine the LCS of two sequences. LCS problems can be solved in $O(n^2)$ running time and memory space, where *n* is the length of the sequences to be dealt with in each case. Generally, MLCS problems are more difficult to solve than LCS ones. Numerous algorithms developed for LCS concerns are inapplicable to MLCS challenges. [7–12], especially large-scale MLCS problems (i.e., problems with numerous and long sequences). As the number and length of sequences rise, the amount of run-time and memory space used exponentially increases owing to the high time and space complexity of $O(n^d)$ [13], where *d* (*d* $\ge$ 2) denotes the number of sequences and *n* denotes the length of sequences.

Similarly, the dominant point-based approach, whose central concept was first introduced by Hakata and Imai [14, 15], is a category of the algorithm for the MLCS problem that is more effective and efficient than its predecessors, which constructs a Directed Acyclic Graph and transforms the problem of finding MLCS into finding the longest path from the source node to the ending node on the DAG graph. It is based on the fact that the vast majority of points in DP-type algorithms’ dynamic tables are irrelevant and that only the critical points, i.e., the so-called dominant points, are required to be calculated and saved [13]. Unquestionably, the dominant point-based approach resulted in a significantly narrower search field than DP-type approaches. It also turns out that considerable performance and memory space reductions are feasible as a consequence of this approach. Subsequently, several versions of the dominant point-based approach have been presented in an effort to further enhance its performance [16–19]. In order to accelerate up a search for the match point’s successors, a special data structure called the successor table was designed by Chen et al. [17] referred to as Fast-LCS. Additionally, Wang et al. [19] described a new method dubbed Quick-DP that employs a divide-and-conquer strategy to accelerate the generation of DAG. In terms of temporal complexity, Quick-DP outperforms Fast-LCS. However, as the number and length of sequences grow, the DAG generated by Fast-LCS and Quick-DP will grow in size. As it turns out, Fast-LCS and Quick-DP often get stuck during the DAG building process. This is because the temporal complexity of both algorithms’ non-dominated sorting approach is $O(N^2)$, where *N* is the number of match points in the DAG, which is substantially more than *n* and *d*.

Recently wang et al. [20] introduced a unique algorithm dubbed Top-MLCS that employs a novel approach for DAG construction and a forward-and-backward topological sorting technique to determine the longest paths in the DAG. Due to the topological sorting technology, this approach has a lower time consumption. Nonetheless, as the size of the DAG grows, topological sorting algorithms consume a significant amount of memory space because they must store the entire DAG, including all match points and paths (i.e., unnecessary match points and non-optimal paths cannot be identified and removed in time). As a result of the memory overflow, they cannot properly tackle the large-scale MLCS problem. Liu et al. [21] offered a character merging algorithm (CMA) that merges consecutively repeated characters, shortens sequences, minimizes the complexity of problems to be handled, and therefore efficiently solves the large-scale MLCS problem. This CMA method is very effective in resolving MLCS with a greater number of repeated characters. In 2020 [22], a new PRDAG model for large-scale MLCS challenges was developed. There are no repeated match points in the PRDAG model, and each match point is allocated a unique path recorder (a key precursor pointer) to keep track of the longest paths connecting the source point to itself. In addition to the optimal algorithm, some excellent heuristic algorithms have been proposed. An algorithm, MLCS-A*, [27] is presented to find an LCS for any given number of sequences. MLCS-A* is a variant of the A* algorithm, a provably optimal best-first search algorithm [28]. But unlike A*, which finds the least-cost path in a graph, MLCS-A* searches in a multidimensional matrix for the longest path corresponding to an LCS. In 2020, a new anytime A* search [26] was proposed to solve the instance problem of various scenarios; apart from providing excellent solutions, the anytime A* search can return proven gaps at almost any time when terminated prematurely. However, with the increase in the number and length of sequences, these strategies still can not cope with the challenges of large-scale MLCS problems.

In this paper, mini-MLCS will be created to assure robust performance while searching for MLCS problems. The primary difference between the proposed algorithm and state-of-the-art algorithms is that mini-MLCS employs a novel path elimination strategy based on lower bound and upper bound estimation to efficiently remove a large number of unnecessary match points and non-optimal paths from a DAG, avoiding the use of non-dominated sorting and topological sorting, which are both extremely time-consuming and require a large amount of memory space. As a result, the size of the created DAG is modest, and mini-MLCS can efficiently identify the longest paths from the DAG to the MLCSs with minimal run-time and memory usage. Our main contributions are as follows:

1. Design a novel branch and bound strategy to eliminate unnecessary paths when constructing DAG graphs. Before obtaining the final MLCS, if we can judge that the currently calculated match point is not the point that constitutes the MLCS, then the path through this point will not be the longest; these are called the non-point and non-optimal paths. Therefore, we do not need to include them in the DAG.
2. Design a smaller DAG (mini-DAG) to prevent the non-dominated and topological sorting during the typical DAG construction process. The proposed branch and bound strategy can eliminate the match points without comparing the match points. It greatly saves time for non-dominated sorting and topological sorting.
3. Propose a fast and efficient algorithm (mini-MLCS) to deal with large-scale sequence problems with lower time and space costs. We design a novel branch and bound graph strategy MLCS algorithm called mini-MLCS and compare it with the state-of-the-art algorithms. The results show that our algorithm is better than these algorithms and is suitable for large-scale MLCS problems.

## Related work

### Definitions of LCS/MLCS problems

#### Definition 1

Let s = $\langle$ $c_1$,$c_2$,...,$c_n$ $\rangle$ represent a sequence on a character set $\Sigma$, where $c_i$ $\in$ $\Sigma$, 1 $\le$ i $\le$ n, $\left| \Sigma \right|$ represents the cardinality of $\Sigma$, and $\left| s\right|$ represents the length of s, i.e.,$\left| s\right|$ = n. If the sequence $s^*$ = $\langle$ $c_{i_1}$,$c_{i_2}$,...,$c_{i_m}$ $\rangle$ fufils 1 $\le$ $i_1$ < $i_2$ < ...< $i_m$ $\le$ n, $s^{*}$ is refered to as a subsequence of s, represented by $s^{*}$ $\in$ sub(s), where sub(s) is the set of all subsequences of s.

Actually, if you eliminate zero or more ordered or unordered characters from a given sequence *s*, the resulting sequence will be shorter than the original sequence *s*. This is referred to as a subsequence of the original sequence *s*.

For example, if $s = \texttt {ACGTA}$ deletes the characters *G* and *T* from the sequence *s*, the resulting sequence $s^* = \texttt {ACA}$ is a subsequence of the sequence *s*.

#### Definition 2

Given a sequence set $Y = \left\{ s_1,s_2,...,s_d \right\}$, where d is the number of sequences contained in Y and d $\ge$ 2, $s_1$, $s_2$,..., $s_d$ on character set $\Sigma$, if there is a sequence $s^*$, which is a subsequence of any sequence in the sequence set Y, then the sequence $s^*$ is called the common subsequence of all sequences in the sequence Y. and if the length of the sequence $s^*$ is the longest of all common subsequences in the set Y, then the sequence $s^*$ is the longest common subsequence of all sequences in the set Y.

For example, $Y = \left\{ s_1 = \texttt {AACGTCGT}, s_2 = \texttt {CGACGTCC}, s_3 = \texttt {GACCGTCT} \right\}$, the existence sequences $s^*_1 = \texttt {ACGTC}$, $s^*_2 = \texttt {AGC}$, ..., $s^*_m = \texttt {C}$ all belong to the common subsequence of $s_1$, $s_2$, $s_3$ in the set *Y*, and $s^*_1$ is the longest among all common subsequences, then the sequence $s^*_1$ is the longest common subsequence of all sequences in the set *Y*.

Usually, there are more than one LCS for given *d* sequences. If $d = 2$, the problem of finding LCS is usually called LCS problem; otherwise, if $d \ge 3$, the problem is called MLCS problem.

#### Definition 3

Give a sequence s = $\langle$ $c_1$,$c_2$,...,$c_n$ $\rangle$, for i = 0,1,...,n, define *pre*(*s*[*i*]) = $\langle$ $c_1$,$c_2$,...,$c_i$ $\rangle$ as the i-th prefix of s and *suf*(*s*[*i*]) = $\langle$ $c_{i+1}$, $c_{i+2}$, ..., $c_n$ $\rangle$ as the (i+1)-th suffix of s(exclude i-th character).

For example, if $s = \texttt {AACGTCGT}$, then $pre(s[5]) = \texttt {AACGT}$ is the 5-th prefix of *s* and $suf(s[5]) = \texttt {CGT}$ is the 6-th suffix of *s*, and *pre*(*s*[0]) is an empty sequence, *suf*(*s*[0]) is an entire sequence.

### Dynamic programming approaches

The DP approach is a time-honored method for solving LCS and MLCS problems [23]. Given *d* sequences of length *n*, $s_1$,$s_2$,...,$s_d$, it will iteratively construct a *d*-dimension score table *L* which have $n^d$ elements, where the element $L[i_1, i_2,..., i_d]$ represents the length of the MLCS of the prefix sequences $pre(s_1[i_1])$, $pre(s_2[i_2])$,..., $pre(s_d[i_d])$, which can be calculated by the following formula [24]:

1 $$\begin{aligned} \begin{array}{l} L\left[ i_{1}, \cdots , i_{d}\right] = \left\{ \begin{array}{ll} 0 &{} \text{ if } \exists i_{j}=0,(1 \le j \le d) \\ L\left[ i_{1}-1, \cdots , i_{d}-1\right] +1 &{} \text { if } s_{1}\left[ i_{1}\right] =\cdots =s_{d}\left[ i_{d}\right] \quad \quad \\ \max ({\bar{L}}) &{} \text { otherwise } \\ \end{array}\right. \end{array} \end{aligned}$$

where $${\bar{L}} = \left\{ L\left[ i_{1}, i_{2}, \cdots ,\left( i_{k}-1\right) , \cdots , i_{d}\right] \mid k=1,2, \cdots , d\right\}$$.

**Fig. 1:**
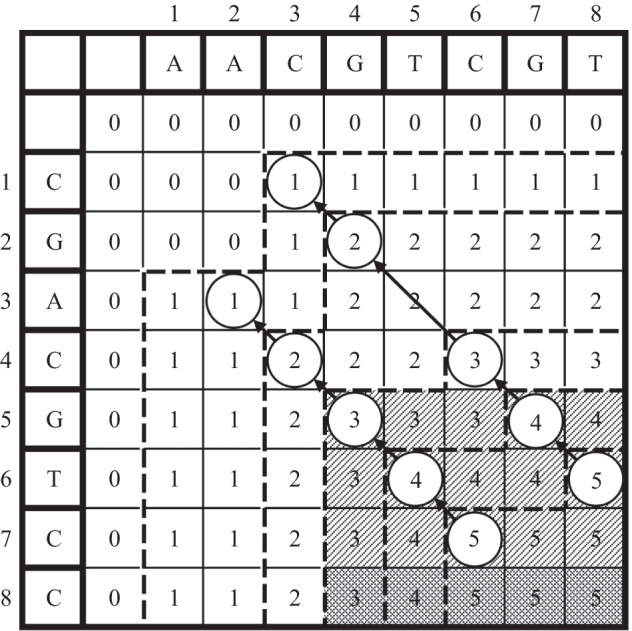
shows the *L* score table for two sequences, $s_1 = \texttt {AACGTCGT}$ and $s_2 = \texttt {CGACGTCC}$. The LCS can be determined by traveling from number 5 to number 1 in the scoring table *L*. And the dominates region may be represented by a shaded portion

After constructing the score table *L*, the MLCS may be calculated by traversing from the bottom-right element *L*[*n*, *n*, ..., *n*] to the top-left element *L*[0, 0, ..., 0]. For example, Fig. 1 illustrates the score table *L* constructed for the sequences $s_1 = \texttt {AACGTCGT}$ and $s_2 = \texttt {CGACGTCC}$, and the LCS for these two sequences is determined by traversing from *L*[8, 8] to *L*[0, 0].

It can be observed from Fig. 1 that for given *d* sequences, and their length is *n*, the DP approach has a time and space complexity of up to $O(n^d)$ [9]. As *d* and *n* expand, these approaches use exponentially more space and time. That is, the scalability of a DP approach is restricted, making it unsuitable for large-scale MLCS problems.

### Dominant point-based approaches

Before delving into the details of the dominant point-based method, we’ll define a few terms.

#### Definition 4

Given a sequence set $Y = \left\{ s_1,s_2,...,s_d \right\}$, where d is the number of sequences contained in Y and d $\ge$ 2, $s_1$, $s_2$,..., $s_d$ on character set $\Sigma$, let $s_i[p_i]$ represent the $p_i$-th character in the left-most sequence $s_i$. If $s_1[p_1]$ = $s_2[p_2]$ = ... = $s_d[p_d]$ = $\sigma$, the vector p = ($p_1$, $p_2$,..., $p_d$) is referred to as a match point for these d sequences. Each match point p = ($p_1$, $p_2$,..., $p_d$) is associated with a distinct symbol $\sigma$. As a result, we often use p = $\sigma$($p_1$, $p_2$,..., $p_d$) to express the match point, where $\sigma$ is the symbol for p, and is represented by Ch(p) = $\sigma$.

For example, if two sequences $s_1 = \texttt {AACGTCGT}$ and $s_2 = \texttt {CGACGTCC}$ are supplied, there are several match points of the form $\sigma (i, j)$. The common character $\sigma$ $\in$ $\Sigma$, connected by a dotted line, corresponds to its indices *i* and *j* in two sequences, i.e.,$s_1[i]$ = $s_2[j]$ = $\sigma$, such as *A*(1, 3), *G*(4, 2). Because $Ch(1,3) = A$, the match point *A*(1, 3) is sometimes abbreviated as (1, 3). Similarly, *G*(4, 2) might be denoted as (4, 2).

#### Definition 5

Given two match points p and q of d sequences on a symbol set T, we say:

1. p = q if $$\forall$$ i (1 $$\le$$ i $$\le$$ d), $$p_{i}=q_{i}$$.
2. p weakly dominates q, if $\forall$ i (1 $\le$ i $\le$ d), $p_{i}$ $\le$ $q_{i}$ and $\exists$ i, $p_{i}$ < $q_{i}$ (denoted by p $\preceq$ q).
3. p dominates q or q is dominated by p, if $\forall$ i (1 $\le$ i $\le$ d), $p_{i}$ < $q_{i}$ (denoted by p $\prec$ q).
4. q is called a successor of p if p $\prec$ q. Further, if there is no match point r to satisfy p $\prec$ r $\prec$ q, then q is called an immediate successor of p.
5. If q is a successor of p, we call p a predecessor of q.

Generally, a match point *p* has no more than $|\Sigma |$ successors.

#### Definition 6

Given a collection of matches P = $\left\{ P_1, P_2, ...,P_m \right\}$, for a match point $P_{j}$ $\in$ P , If $\lnot$ $\exists$ $P_{i}$ $\preceq$ $P_{j}$, 1 $\le$ i, j $\le$ m, i $\ne$ j, $P_{j}$ is called a non-dominated point (dominant point for short) on P. All of dominant points on P form the dominant set of P .

The dominant point-based approaches are based on constructing a direct acyclic graph (DAG). Their organizational structure is as follows. To begin, given *d* sequences, the graph’s source point is defined as a *d*-dimensional point *O*(0, 0...0). This point has no input edge, so its in-degree is 0. The source point’s level is defined as level 0. Following that, we identify all successor points of the present point *O* and create a directed edge connecting it to every one of its successors. The successor points’ level is defined as level 1. Non-dominated sorting is used to compute the set of all dominated points on *level*-1. Then, for each non-dominated point on *level*-1, we identify all successor points, create an edge connecting each non-dominated point on *level*-1 to every successor, and designate the level of all these successors as level 2. We use non-dominated sorting to identify all dominated points on *level*-2. This procedure is continued until no additional successor points are formed, at which time the DAG building is complete. If a point without a successor, it is defined as the ending point ($\infty$,$\infty$,...,$\infty$). When the DAG is established, the LCS/MLCS is formed by the character sequence represented by the points along the longest path from the source point to the ending point. Thus, the LCS/MLCS problem’s primary problem is how to create the DAG.

**Fig. 2:**
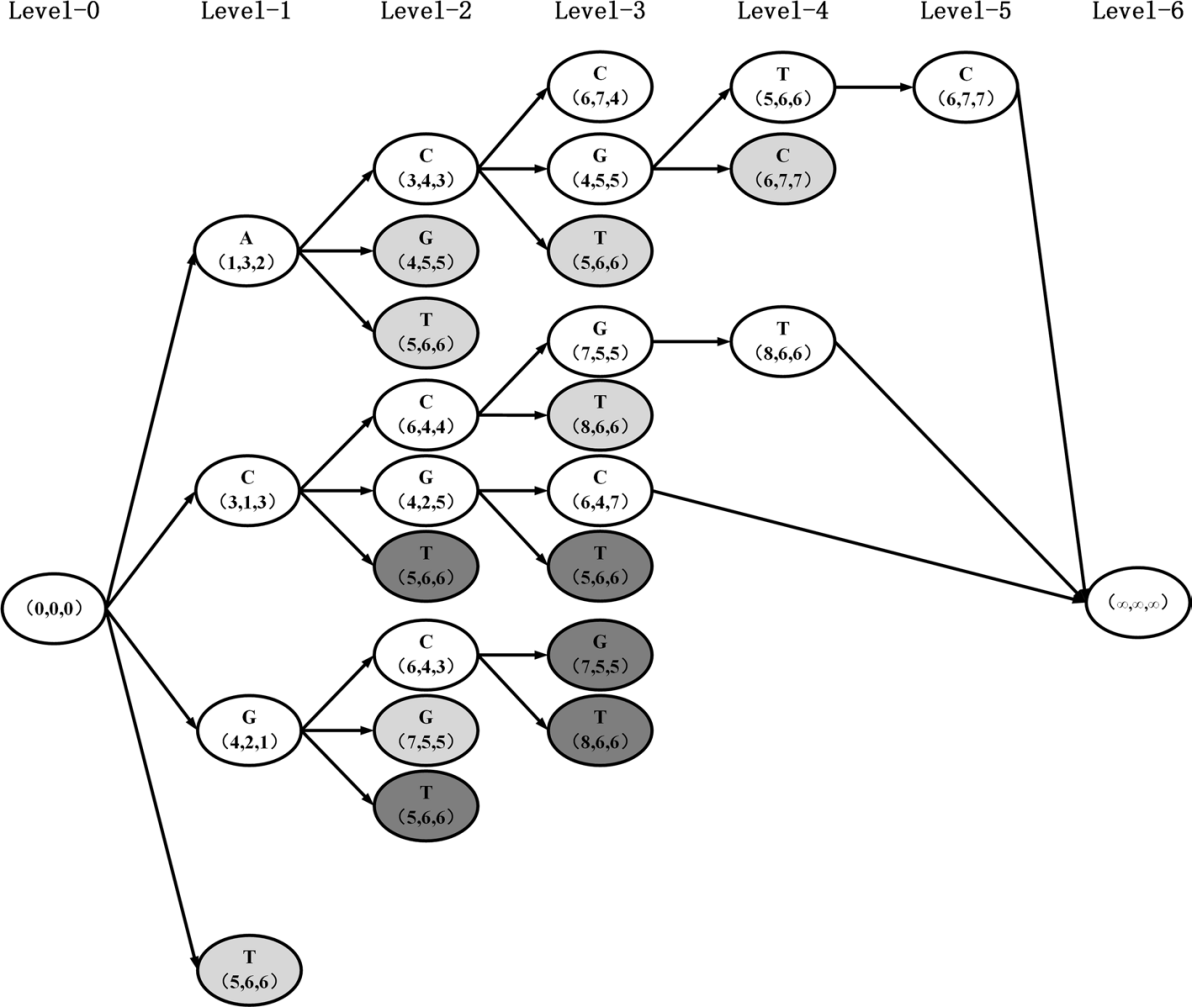
The DAG is constructed for three sequences, $s_1 = \texttt {AACGTCGT}$, $s_2 = \texttt {CGACGTCC}$ and $s_3 = \texttt {GACCGTCT}$, with black and gray nodes representing repeated and dominated nodes, respectively

For example, as shown in Fig. 2, the sequences $s_1 = \texttt {AACGTCGT}$, $s_2 = \texttt {CGACGTCC}$ and $s_3 = \texttt {GACCGTCT}$, the MLCS is generated using the dominant points-based approach.

1. Initialization.
  Set the source node *O*(0, 0, 0) and the ending node ($\infty$,$\infty$,$\infty$).
2. DAG construction on level 1.
  For point *O*(0, 0, 0) on $D_0$, find all of its successors: *A*(1, 3, 2), *C*(3, 1, 3), *G*(4, 2, 1) and *T*(5, 6, 6), and add a direct edge from point *O*(0, 0, 0) to each one of them. These successors are all level-0 successors and all level-1 points. Put them in $L_1$’s set. The dominated point *T*(5, 6, 6) in set $L_1$ is removed using non-dominated sorting (notice that *A*(1, 3, 2) $\prec$ *T*(5, 6, 6), *T*(5, 6, 6) is a dominated point). Set $D_1$ $=$ $\left\{ A(1, 3, 2), C(3, 1, 3), G(4, 2, 1) \right\}$, and *k* to *k* $+$ 1.
3. DAG construction on level 2.
  For each point in $D_1$ $=$ $\left\{ A(1,3,2), C(3,1,3), G(4,2,1) \right\}$, find all of its successors. *C*(3, 4, 3), *G*(4, 5, 5), and *T*(5, 6, 6) are all successors to point *A*(1, 3, 2) $\in$ $D_1$ and are included in $L_2$. Create a direct edge connecting point *A*(1, 3, 2) to each one of its successors. For point *C*(3, 1, 3), *C*(6, 4, 4), *G*(4, 2, 5), and *T*(5, 6, 6) are all its successors and are included in $L_2$. Add a direct edge from *C*(3, 1, 3) to each of its successors. For point *G*(4, 2, 1), *C*(6, 4, 3), *G*(7, 5, 5), and *T*(5, 6, 6) are all its successors and add a direct edge from *G*(4, 3, 2) to each of its successors, put these successors into $L_2$. Eliminate the redundant successors in $L_2$. The dominated points *G*(4, 5, 5), *T*(5, 6, 6) and *G*(7, 5, 5) are deleted using the non-dominated sorting on $L_2$. Allow $D_2$ = $\left\{ C(3, 4, 3), C(6, 4, 4), G(4, 2, 5), C(6, 4, 3) \right\}$, and *k* to *k* $+$ 1.
4. Repeat step 3 until no successor exists for all points in the set $D_k$. Then substitute $(\infty , \infty , \infty )$ for the points in $D_k$, and the DAG construction is complete.

As seen above, the dominant point-based techniques have the following significant disadvantages:

1. Each level may contain numerous repeated match points and dominated points. (e.g., *T*(5, 6, 6) appears three times in $L_2$ and three points *G*(4, 5, 5), *T*(5, 6, 6), *G*(7, 5, 5) are dominated points), and a match point appearing in one level may appear numerous times in subsequent levels (e.g., *T*(5, 6, 6) appears in $L_1$-$L_4$) and is only useful in the final level. Thus, the created DAG will be very large, to the point that the computer will run out of memory to hold it.
2. The non-dominated sorting approach will need a significant amount of work to obtain $D_k$. It has an $O(d{N_{k}}^2)$ time complexity at level *k*, where $N_k$ is the number of match points in $L_k$ and *d* represents the number of sequences. Note that $N_k$ will be really huge (in the worst-case case, $N_k = |\Sigma |^k$ rises exponentially as level *k* increases). Thus, when *n*, *d*, and $\left| \Sigma \right|$ are big, i.e., when the MLCS issue becomes a large-scale problem, the non-dominated sorting approach becomes very time-consuming.

Fast-LCS [17] and Quick-DP [19] are two representative algorithms of this kind.

## The proposed mini-MLCS

### The main framework of mini-MLCS

As previously stated, existing approaches cannot address large-scale MLCS problems owing to their enormous time and space requirements [22]. The underlying reason behind this is that as the number *d* and length *n* of sequences grow, non-dominant sorting and topological sorting will spend a lot of time on the comparison between match points. As it turns out, the computing time and storage space requirements surpass the maximum limits. To address these problems, the proposed mini-MLCS rapidly finds unnecessary match points and non-optimal paths during DAG building and then eliminates them in time to limit the DAG’s size.

To be precise, in order to get the final MLCS of sequences, mini-MLCS first designed a strategy to quickly predict the genuine MLCS *R*’s lower bound *Lower*(*R*). Then, before deciding whether or not to include match point *p* in the DAG, mini-MLCS calculates an upper bound $Upper(p, \infty )$ on the length of any path from the match point *p* to the ending match point. Assuming that the true distance between *p* and the ending point is the *distance*(*p*), the obtained upper bound $Upper(p,\infty )$ should be greater than or equal to *distance*(*p*). Finally, determine whether the estimated distance from the starting point to the ending point is less than *Lower*(*R*) (Note that the distance from the starting point O to *p* is the current level value *level*(*p*) when the DAG calculates to *p*). If $Upper(O,p,\infty )$ = *level*(*p*) + $Upper(p,\infty )$ < *Lower*(*R*), then no path via *p* is the longest path. As a result, *p* is an unnecessary match point, and all paths going through it are not the longest in the DAG. Based on this discovery, all unnecessary match points and non-optimal paths may be deleted immediately.

### Estimation of the lower bound *Lower*(*R*) in a short time

We do not really know the genuine length of MLCS *R* until we receive it, but we can gain a lowest bound *Lower*(*R*) by generating an estimated MLCS. Then the length of this estimated MLCS is a lower bound on *R*, The longer the length of the estimated MLCS, the more closely it resembles *R*. Our objective is to find an estimated MLCS as rapidly as feasible. A rapid heuristic strategy for calculating the lower bound *Lower*(*R*) is designed based on these concepts. The critical stages are listed below.

For a *d*-dimensional match point *p* $=$ ($p_1$, $p_2$,... , $p_d$), *max*(*p*) represents the largest number in the match point *p*, *min*(*p*) represents the smallest number in the match point *p*, and $\varphi (p) = max(p) - min(p)$ represents the largest position offset of *d* sequences in the match point *p*. Among all match points at each level, we pick up the first *t* smallest $\varphi ()$. Because it can be observed from Fig. 1, the match point with the smaller $\varphi ()$ tends to contain a larger dominates regions than points of larger $\varphi ()$, and larger dominates regions may contain more match points, so the smaller $\varphi ()$ is more likely to be the match point that constitutes the longest common subsequence than the match point with the larger $\varphi ()$. For example, in Fig. 1, *C*(3, 4) contains more dominates regions than *C*(3, 7), so *C*(3, 4) is more likely to form the longest common subsequence. Based on this idea, two strategies are proposed to get an accurate lower bound.

**Strategy 1:** Assigning a small initial value to *t* in the DAG construction process means that *t* match points with the smallest $\varphi ()$ value are selected in each level of the DAG. The role of *t* is to reduce the search space and get a suitable *Lower*(*R*) at a faster speed. In this way we will get an initial *Lower*(*R*). Next, add a certain step length $\mu$ to *t* each time, so that $t = t + \mu$, and continue to construct the DAG to calculate the *Lower*(*R*). Update *Lower*(*R*) if it changes, if there is no change in the *Lower*(*R*) for more than $\tau$ (we define it by ourselves) times, then it can be considered that a more accurate *Lower*(*R*) has been obtained.

The following is an example of calculating the lower bound.

Assume *t* is a small positive integer (for example, $t = 4$) and *D* is a collection of *t* randomly chosen match points with the first *t* lowest values of $\varphi ()$

1. Initialization: Set $O = (0,0,...,0)$ as the first chosen match point, i.e., $D = \left\{ O \right\}$, and set $Lower(R) = 0$.
2. Update *Lower*(*R*): Pick up *t* successors with the first *t* lowest values of $\varphi ()$ from all successors in *D* (if there are only $\beta$ successors with $\beta$ $\le$ *t*, then let $t =$ $\beta$). Update *D* by deleting all of its existing elements and inserting the chosen match points, and letting $Lower(R) = Lower(R) + 1$.
3. Return *Lower*(*R*) if *D* is empty; otherwise, proceed to step 2.

**Fig. 3:**
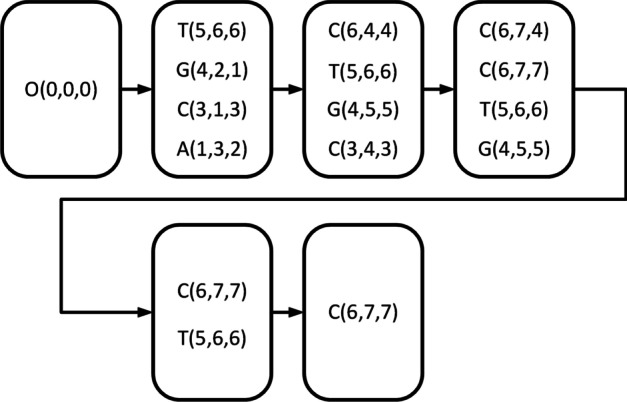
The process of estimating a lower bound *Lower*(*R*) of the length of the longest paths in DAG

In Fig. 3, we utilize the previous example to demonstrate the method. $Lower(R) = 0$ at first, and $D = O$. *O* has four successors (1, 3, 2), (3, 1, 3), (4, 2, 1), (5, 6, 6). because $t =$ 4, all these successors are selected. Thus, we update *D* by $D = \left\{ (1,3,2),(3,1,3),(4,2,1),(5,6,6) \right\}$ and set $Lower(R)= Lower(R)+ 1$. Calculate the successors for each match point in *D*, and there are 10 successors in total (in Fig. 2, in order to find the lower bound as soon as possible, point filtering is not carried out, that is, all filtered points will be included, including the successor point *C*(6, 7, 7) of *T*(5, 6, 6)), with four successors (3, 4, 3),(4, 5, 5), (5, 6, 6), (6, 4, 4) with the first four smallest $\varphi ()$ values selected (note that the smallest $\varphi ()$ value match point is (3, 4, 3), (4, 5, 5), (5, 6, 6) and the second smallest $\varphi ()$ value match point has two: (6, 4, 4) and (7, 5, 5) with the same $\varphi ()$ value. In this scenario, we merely need to choose one at random from (6, 4, 4) and (7, 5, 5) (assuming (6, 4, 4) is chosen) and update *D* by $D =$ $\left\{ (3,4,3),(4,5,5,),(5,6,6),(6,4,4) \right\}$ and update *Lower*(*R*) by $Lower(R) = 2$. Similarly, with $Lower(R) = 3$, $D =$ $\left\{ (4,5,5),(5,6,6),(6,7,7),(6,7,4) \right\}$. When $Lower(R) = 4$, the appropriate $D =$ $\left\{ (6,7,7),(5,6,6) \right\}$. Finally, we obtain $Lower(R) = 5$ and the appropriate $D =$ $\left\{ (6,7,7) \right\}$.

### Estimation of the upper bound $Upper(O,p,\infty )$ with efficiency

Assuming that *p* is a current point on the DAG and that we want to know the lengths of all paths from *O* to the ending match point that passes through *p*. However, the lengths of these paths are unknown until they are constructed. But if we can estimate an upper bound $Upper(O,p,\infty )$ on the lengths of these paths and know that it is less than the lower bound *Lower*(*R*) (i.e., $Upper(O,p,\infty )$ < *Lower*(*R*)), then we can conclude that these paths via *p* are not the longest paths and can be removed from the DAG. In this manner, the new DAG will be far smaller than the previous ones.

Notably, the DAG’s current match point *p* has been established, and the length of the longest path from *O* to *p* may be determined. Indeed, it is the DAG level of *p* (denoted by *level*(*p*)).

Additionally, the genuine length of the longest path *distance*(*p*) between the current match point *p* and the ending match point is generally unknown. A possible method is to estimate the upper bound $Upper(O,p,\infty )$. Then $Upper(O,p,\infty )= level(p) + Upper(p,\infty )$ is the upper bound on the length of any path via *p*. In the following, we will design some strategies for rapidly estimating $Upper(p,\infty )$ and bringing it as near to the real value $distance(p,\infty )$ as feasible (i.e., make it as small as possible).

Given *d* sequences $s_1$, $s_2$,...,$s_d$ on a character set and a match point $p =$ ($p_1$, $p_2$,..., $p_d$), the following conclusion is obtained:

#### Theorem 1

For each longest path between match point p = ($p_1$, $p_2$,..., $p_d$) and the ending match point, and n is the length of the sequence corresponding to max(p). Hence Upper(p,$\infty$) = n - max(p) is an upper bound on the length of any longest path between match points p = ($p_1$, $p_2$,..., $p_d$) and the ending match point $\infty$, and Upper(O,p,$\infty$) = level(p) + n - max(p) is an upper bound of the length of the longest path from O to $\infty$ through p.

#### *Proof*

Denote $\xi$ $= n - max(p)$. Obviously, *distance*(*p*) is equal to the longest common subsequence of sequence $suf(s_i[p_i])(1 \le i \le d)$, $distance(p) \le \xi$ because for the sequence corresponding to *max*(*p*) there are at most $\xi$ character after it. Therefore $Upper(p,\infty ) = n - max(p)$, namely $Upper(O,p,\infty ) = level(p) + n - max(p)$.

**Fig. 4:**
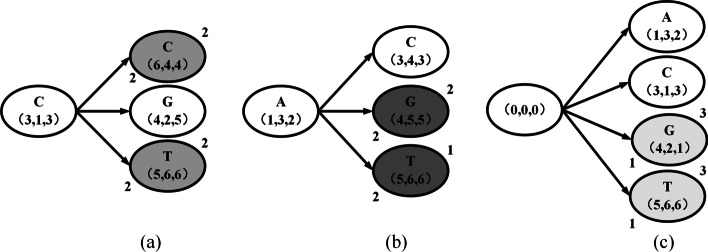
**a**, **b** and **c** show the processes of using Theorem 1, Strategy 2, and Strategy 3 to removing unnecessary points, respectively. And grey point, dark grey point and light grey point represent unnecessary points in a, b and c, respectively

Some extensions can be developed on the basis of Theorem 1. Select the first $\delta$ *max*(*p*) of match point *p*, then compute the LCS of sequences ${suf(s_i[max_i(p)])\ (}1 \le i \le \delta )$. and use it as the upper bound of *p*. The upper bound obtained will undoubtedly be closer to *distance*(*p*) than the upper bound obtained by Theorem 1, but it will take longer to calculate each match point.

Let us analyze the aforementioned example in Fig. 2 in further depth to demonstrate the strategy for identifying unnecessary match points. The MLCS’s estimated lower bound is known, i.e., $Lower(R) = 5$, shown in Fig. 3. According to Theorem 1, $upper(p,\infty )$ can be estimated, and *level*(*p*) can be achieved during the DAG construction process, shown in Fig. 4a.

The match point *C*(3, 1, 3)’s *level*(*C*) is defined as 1. *C*(3, 1, 3) is followed by three successors: *C*(6, 4, 4), *G*(4, 2, 5) and *T*(5, 6, 6). Set *level*(*p*) equal to 2 for each of its successors, i.e., $level(C(6, 4, 4)) = level(G(4, 2, 5)) = level(T(5, 6, 6)) = 2$, because the length of the longest paths from the starting match point to each of the successors is 1, and $Lower(R) = 5$, shown in Fig. 3. According to Theorem 1, $Upper(C(6, 4, 4),\infty ) = 8 - max(C(6, 4, 4)) = 2$, $Upper(C(4, 2, 5),\infty ) = 8 - max(C(4, 2, 5)) = 3$ and $Upper(C(5, 6, 6),\infty ) = 8 - max(C(5, 6, 6)) = 2$. So as seen in Fig. 4a, match point *C*(6, 4, 4) and *T*(5, 6, 6) are clearly identified as unnecessary match points since they meet

$$Upper(O,p, \infty ) = level(p) + Upper(p, \infty )$$ < *Lower*(*R*)

and none of the paths (branches) linking *O* and them will be included in the DAG.

In addition to the methods for finding the upper bound introduced above, the literatures [26] and [27] mentioned two more compact strategies for finding the upper bound. They build vectors in the preprocessing stage and then use them to find the upper bound for each match point in the DAG construction stage. The specific details are expanded in strategy 2 and strategy 3.

**Strategy 2:** Let ${num_{s_{i}}^c}$ represent the number of the character *c* in sequence ${s_i\ (}1 \le i \le d)$. For each longest path between match point *p* = ($p_1$, $p_2$,..., $p_d$) and the ending match point. The following conclusions can be drawn, for any *c* in $\Sigma$, number of *c* in the sequence on the path from *p* to the end node will not exceed $min\left\{ num_{suf(s_1[p_1])}^c,num_{suf(s_2[p_2])}^c,...,num_{suf(s_d[p_d])}^c\right\}$.

Hence,

2 $$\begin{aligned} \begin{array}{c} Upper_{(p, \infty )}=\sum \limits _{c \in \Sigma } \min \left\{ num_{suf(s_1[p_1])}^c,num_{suf(s_2[p_2])}^c,...,num_{suf(s_d[p_d])}^c\right\} \end{array} \end{aligned}$$

Figure 4b shows an application of strategy 2. The match point *A*(1, 3, 2) has three successors in *level*-2, namely *C*(3, 4, 3), *G*(4, 5, 5) and *T*(5, 6, 6). For *G*(4, 5, 5), $suf(s_1[4]) = \texttt {TCGT}$, $suf(s_2[5]) = \texttt {TCC}$ and $suf(s_3[5]) = \texttt {TCT}$. $Upper(G(4,5,5),\infty ) = \sum \limits _{c \in \Sigma } \min \left\{ num_{suf(s_1[p_1])}^c,num_{suf(s_2[p_2])}^c,num_{suf(s_3[p_3])}^c\right\} = 1 + 1 + 0 + 0 = 2$, because for $\texttt {C}$ and $\texttt {T}$, their minimum number of occurrences in $suf(s_1[4])$, $suf(s_2[5])$ and $suf(s_3[5])$ is once, $\texttt {A}$ and $\texttt {G}$ appears at least 0 times in $suf(s_1[4])$, $suf(s_2[5])$ and $suf(s_3[5])$. $Upper(O,G(4,5,5),\infty ) = level(G(4,5,5)) + Upper(G(4,5,5),\infty ) = 2 + 2$ < *Lower*(*R*), match point *G*(4, 5, 5) is identified as unnecessary point and none of the paths (branches) linking *O* and *G*(4, 5, 5) will be included in the DAG.

**Strategy 3:** For each longest path between match point *p* = ($p_1$, $p_2$,..., $p_d$) and the ending match point and a vector $m_{i}$, where $m_{i}[p_{i}, p_{i+1}]$ with $p_{i} = 1, . . . , |s_{i}|$ and $p_{i+1} = 1, . . . , |s_{i+1}|$, stores the length of the LCS of strings $suf(s_i[p_{i}])$ and $suf(s_{i+1}[p_{i+1}])$.

Hence,

3 $$\begin{aligned} \begin{array}{c} Upper_{(p, \infty )}=\min \limits _{i=1, \ldots , d-1} m_i[p_{i},p_{i+1}] \end{array} \end{aligned}$$

Figure 4c shows an application of strategy 3. For match point *G*(4, 2, 1) on *level*-1, $suf(s_1[4]) = \texttt {TCGT}$, $suf(s_2[2]) = \texttt {ACGTCC}$ and $suf(s_3[1]) = \texttt {ACCGTCT}$. The result is $Upper(G(4,2,1),\infty )$ $= min(3,5) = 3$, it is the minimum value of LCS of $suf(s_1[4])$ and $suf(s_2[2])$ and LCS of $suf(s_2[2])$ and $suf(s_3[1])$, namely $Upper(O, G(4,2,1), \infty ) = level(G(4,2,1)) + Upper(G(4,2,1),\infty ) = 3 + 1 = 4 < Lower(R)$, match point *G*(4, 2, 1) is identified as unnecessary point and none of the paths (branches) linking *O* and *G*(4, 2, 1) will be included in the DAG.

For strategies 2 and 3, when the scale of *n* and *d* is particularly large, it will take a long time to preprocess. Therefore, in order to reduce the preprocessing time, we do not need to apply all sequences to the preprocessing, but choose $\delta$ sequences as different as possible. In this way, we can get the pretreatment results in a shorter time.

Fortunately, Wang et al. [25] define a metric for comparing two sequences. This metric is referred to as the diversity metric.

Let ${num_{s_{i}}^c}$ represent the number of the character *c* in sequence $s_i$. Note that the greater the value of $\left| {num_{s_{i}}^c} - {num_{s_{j}}^c}\right|$, the more diverse $s_i$ and $s_j$ are, and the greater the difference between these two sequences. By considering this factor, the diversity between $s_i$ and $s_j$ is defined as:

4 $$\begin{aligned} diversity\left( s_{i}, {s}_{j}\right) =\sum _{c \in \Sigma } \frac{num_{s_{i}}^c}{|{s_i}|}\left| num_{s_{i}}^c-num_{{s}_{j}}^c\right| \quad \quad \quad \end{aligned}$$

Here are a few instances that illustrate the above.

We can choose the second sequence with greater diversity using sequence $s_1$ based on the diversity measure.

$$s_1 = \texttt {AACGTCGT}$$.

$$s_2 = \texttt {CGACGTCC}$$.

$s_3 = \texttt {GACCGTCT}$, we choose $s_1$ = $s_i$, then we count how many times each character appears in these sequences.

$${num_{s_{1}}^A} = 2$$, $${num_{s_{1}}^C} = 2$$, $${num_{s_{1}}^G} = 2$$, $${num_{s_{1}}^T} = 2$$

$${num_{s_{2}}^A} = 1$$, $${num_{s_{2}}^C} = 4$$, $${num_{s_{2}}^G} = 2$$, $${num_{s_{2}}^T} = 1$$

$${num_{s_{3}}^A} = 1$$, $${num_{s_{3}}^C} = 3$$, $${num_{s_{3}}^G} = 2$$, $${num_{s_{3}}^T} = 2$$

$$diversity\left( s_{1}, {s}_{2}\right) = \frac{2}{8}|2 - 1| + \frac{2}{8}|2 - 4| + \frac{2}{8}|2 - 2| + \frac{2}{8}|2 - 1| = \frac{3}{4}$$

$$diversity\left( s_{1}, {s}_{2}\right) = \frac{2}{8}|2 - 1| + \frac{2}{8}|2 - 3| + \frac{2}{8}|2 - 2| + \frac{2}{8}|2 - 2| = \frac{1}{2}$$

As a result, we conclude that $s_1$ and $s_2$ are more dissimilar than $s_1$ and $s_3$.

Therefore, when the scale of the sequence is large, we choose $s_1 = s_i$ and pick up $\delta$ $(\delta \ll d)$ most different(contains $s_1$ sequence) sequences from the given *d* sequences using the formula (4). In this way, we greatly reduce the preprocessing time of strategies 2 and 3.

### Construct mini-DAG

Based on the above branch elimination approach, we construct the mini-DAG level by level. First, level zero $D_0$ consists of merely the beginning match point *O*, and then level 1 through level *R*, represented by $D_1$, $D_2$,..., $D_R$, respectively, are consecutively created, where *R* denotes the length of the final MLCS. To minimize the time and space, we merely create and store one level each time.

After $D_k$ is constructed (currently, $D_0$ is constructed), the following procedures can be taken to build $D_{k+1}$:

1. Select every match point $p \in D_k$, search its successorset *succ*(*p*).
2. For each successor $q \in succ(p)$, set the level of *q* (i.e.,the length of the current longest path from *O* to *q*) as $level(q) = k + 1$.
3. Identify whether *q* is a useless match point according to Theorem 1, Strategy 2 and Strategy 3. If yes, do not put *q* in DAG, go to step 5. Otherwise, put *q* into $D_{k+1}$.
4. Add a directed edge from *p* to *q* in DAG.
5. If successors of all match points in $D_k$ have been checked, the construction of $D_{k+1}$ is finished. Otherwise, go to step 1.

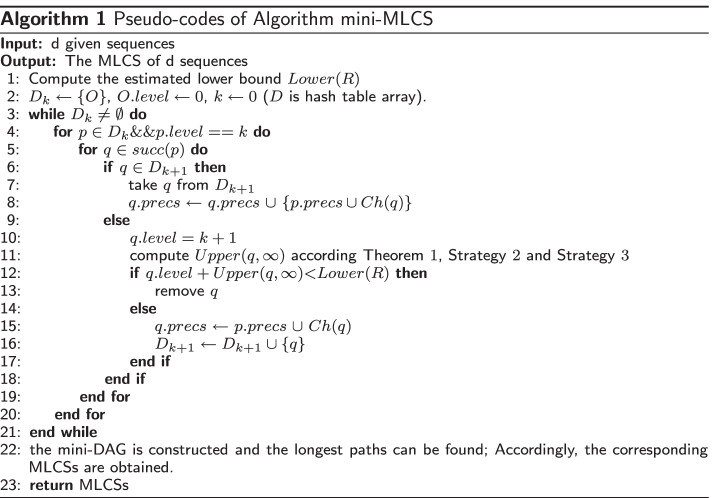


### Mini-MLCS algorithm

The pseudo-codes of algorithm mini-MLCS are presented in Algorithm 1 in order to describe the new algorithm in detail.

At the beginning, the estimated lower bound *Lower*(*R*) is calculated. The proposed algorithm’s key steps are lines 3 $\sim$ lines 21, which explain how a mini-DAG is built level by level. *Ch*(*q*) in the lines 8 means the character represented by the match point *q*, which is described in Definition 4, and *q*.*precs* represents the longest common subsequence from the beginning match point *O* to the current point *q*. Finally, from the mini-DAG, the longest paths corresponding to MLCSs may be obtained, and all MLCSs will be returned in lines 22 $\sim$ lines 23.

## Time and space complexity

### Mini-MLCS time complexity

In order to show the efficiency of Algorithm 1 compared with other algorithms, the time complexity of the proposed Algorithm 1 and the comparison algorithm are shown here. First, the length of the sequences is denoted by *n*, and *d* represents the number of sequences. In the initialization, we built the Successor Table that was proposed by Fast-LCS so that we could rapidly discover the successor nodes of a point with $O(d |\Sigma | n)$ [17]. Second, we estimate the time cost to find successor nodes and add them to the Vector Hash Table. Use *N* to represent the whole collection of points in the mini-DAG and the time complexity is *O*(|*N*|). Finally, in mini-DAG, we use *E* to represent the whole collection of edges, and the time complexity is *O*(|*E*|). For strategy 1, the time complexity of building a DAG once and finding it’s lower bound is $O(d|\Sigma ||MLCS|t)$. Then calculate the time complexity of the upper bound. In Theorem 1, the time complexity of finding the upper bound is *O*(*d*). In the pretreatment phase (before the mini-DAG construction), we choose the $\delta$ sequence from *d* sequence by formula (4) and apply it to strategies 2 and 3. The preprocessing results are stored by using appropriate data structures, we can compute the $Upper(p,\infty )$ of strategy 2 at any match point quickly, the time complexity is $O(\delta |\Sigma |)$ for each match point. And strategy 3 in the preprocessing phase, it takes $O(\delta n^{2})$. Therefore, considering the worst case, the time complexity of mini-MLCS is $O(d |\Sigma | n)$ + $O(d|\Sigma ||MLCS|t)$ + $O(\delta n^{2})$ + *O*(|*E*|) + *O*(|*N*|). Since $O(d |\Sigma | n) \ll O(\delta n^{2})$, $O(d|\Sigma ||MLCS|t) \ll O(\delta n^{2})$, *O*(|*N*|) = *O*(|*E*|) and $O(\delta n^{2})$ < *O*(|*N*|), the time complexity of our proposed Algorithm 1 is *O*(|*N*|).

For the compared algorithms, Quick-DP takes $O\left( d(\log n)^{d-2}\left| N_{Q}\right| \right)$ [19], where $N_{Q}$ is the set of points in the DAG constructed by Quick-DP, and the time complexity of Top-MLCS is $O(|N_{T}|)$ [20], where $N_{T}$ is the set of points in the ICSG constructed by Top-MLCS. It should note that, due to the lack of a reasonable scheme to reduce the search space, the DAG constructed by Top-MLCS is much larger than those constructed by Quick-DP and mini-MLCS, and DAG constructed by Quick-DP is larger than that constructed by mini-MLCS, i.e., $|N_{T}|$ $\gg$ $|N_{Q}|$ > |*N*|. But Quick-DP uses the time-consuming non-dominated sorting method to reduce the search space, so $O\left( d(\log n)^{d-2}\left| N_{Q}\right| \right)$ > $O(|N_{T}|)$. Thus, $O\left( d(\log n)^{d-2}\left| N_{Q}\right| \right)$ > $O(|N_{T}|)$ $\gg$ *O*(|*N*|).

### Mini-MLCS space complexity

Next, we calculate the space complexity of Algorithm 1. The space consumed by the Successor Table is $O(d |\Sigma | n)$; for strategy 2, the space complexity is $O(\delta |\Sigma | n)$, the space complexity of strategy 3 is at most $O(\delta n^2)$, but the space spent by storing points is *O*(*d*|*N*|). And the storing edge takes up *O*(|*E*|) area. Since $O(d|\Sigma |n) \ll O(d|N|) + O(|E|)$, $O(\delta |\Sigma | n) \ll O(d|N|) + O(|E|)$, $O(d|N|) = O(|E|)$ and $O(\delta n^2) \ll O(d|N|) + O(|E|)$, the space complexity of Algorithm 1 is *O*(*d*|*N*|). The space complexity of Quick-DP and Top-MLCS can be expressed as $O(d|N_{Q}|)$ [19] and $O(d|N_{T}|)$ [20], respectively. For $|N_{T}|$ > $|N_{Q}|$ > |*N*|, we can deduce that our mini-MLCS algorithm has lower space complexity than two compared algorithms due to the use of the branch and bound strategy.

## Experiments and analysis

### Experimental setups and compared algorithms

To illustrate mini-MLCS’s performance on large-scale MLCS problems, we conduct experiments comparing it to four state-of-the-art algorithms Fast-LCS [17], Quick-DP [19], Top-MLCS [20], and A* search [26]. All experiments are conducted on a server equipped with four Intel(R) Xeon(R) E5-2640 2.40 GHz ten-core CPUs, 160 GB RAM, 4 NVidia Tesla K40 graphics cards, and 1.1TB of disc space. Ubuntu 16.04 is the operating system. All algorithms are written in Eclipse and compiled using C and C++ code. Biological sequences from NCBI http://www.ncbi.nlm.nih.gov/nuccore/110645304?report = fasta are selected as the test sets. This is the complete genome sequence of Pseudomonas aeruginosa PAO1, and the experimental data will be randomly selected from this genome. The related literature [26] on the LCS problem offers a public benchmark sets for the LCS problem. The BL benchmark [26] consists of 450 problem instances grouped by different values for the number of input strings (*d*), the maximum length of the input strings (*n*), and the alphabet size ($|\Sigma |$). For each combination of *d*, *n*, and $|\Sigma |$ the set offers ten instances generated uniformly at random. We conduct the following four types of experiments. And these results are summarized in Tables 1, 2, 3 and 4.

For the first kind of experiment, we fixed the sequence length to 120 and performed trials on 9 examples with a sequence count ranging from 10,000 to 50000. For the second kind of experiment, we limit the number of sequences to 20,000 and run the experiment on sixteen cases with sequence lengths ranging from 90 to 120. For the third kind of experiments, we extract instances with sequence length of 100 from the BL set and obtained the average results in Table 3. For the last kind of experiments, to show the estimate method’s robustness, we evaluate the effects of *t* on *Lower*(*R*) by changing the values of *t* through the experiments.

Column 1 in Tables 1 and 3 indicates the total number of DNA sequences; column 1 in Tables 2 and 4 indicates the total length of DNA sequences; column 2 in Tables 1, 2 and 4 indicates the total length *R* of MLCS in the test sequences. In Tables 1, 2 and 4, columns 3 to 6 provide the mean running time(s)/memory(GB) for mini-MLCS, Top-MLCS, Quick-DP and Fast-MLCS respectively. Bold face numbers denote the data set’s lowest running time, and ’+’ indicates that results are not obtained when the running time exceeds 5000 s. For Table 3, ’−’ indicates that the running memory exceeds 32GB and the result cannot be obtained.

### Experimental results and analysis

As seen in Table 1, the Fast-LCS algorithm consistently fails to handle DNA sequences with a length of 10,000 to 50,000, owing to its extraordinarily lengthy runtime. At the same time the Quick-DP algorithm is likewise incapable of dealing with DNA sequences with a length of between 10,000 and 25,000. The results indicate that the difficulty of the tasks does not increase as the number of sequences increases. This is because, for a fixed length of sequences, increasing the number of sequences increases the cost of searching all MLCS, but once the number of sequences reaches a certain level, both the number and length of MLCS drop. As a result, the cost of searching the MLCS is reduced. The primary reason these two algorithms take so long is that they require too much time for non-dominated sorting (note that as the number of sequences increases, non-dominated sorting requires a significant amount of time and space), whereas Top-MLCS and mini-MLCS do not require non-dominated sorting and thus take much less time.

**Table 1:** The run time (s)/memory (GB) consumed by the compared algorithms on DNA sequences with length fixed to 120

Number of sequences	*R*	DNA($|\Sigma | = 4$)			
		mini-MLCS	Top-MLCS	Quick-DP	Fast-MLCS
10,000	15	${\textbf {777.1/8.1}}$	1245.7/31.8	$+$	$+$
15,000	14	${\textbf {966.3/14.7}}$	1499.2/17.2	$+$	$+$
20,000	13	${\textbf {1151.1/12.3}}$	1362.8/17.6	$+$	$+$
25,000	13	${\textbf {1162.7/11.1}}$	1225.1/35.8	$+$	$+$
30,000	11	${\textbf {173.9/1.8}}$	226.1/2.9	515.5/5.1	$+$
35,000	11	${\textbf {146.3/1.9}}$	403.0/3.9	590.6/5.1	$+$
40,000	11	${\textbf {234.4/1.8}}$	256.1/3.1	432.6/5.5	$+$
45,000	11	${\textbf {174.9/2.1}}$	393.7/3.4	480.2/5.4	$+$
50,000	11	${\textbf {195.9/2.1}}$	298.8/3.6	448.1/6.6	$+$

The bold values represent the minimum running time and minimum memory of all the algorithms in the table on the dataset

**Table 2:** The run time (s)/memory (GB) consumed by the compared algorithms on DNA sequences with number fixed to 20,000

Length of sequences	*R*	DNA($|\Sigma | = 4$)			
		mini-MLCS	Top-MLCS	Quick-DP	Fast-MLCS
90	8	3.7/0.2	4.8/0.1	${\textbf {2.8/0.1}}$	38.84/0.8
95	9	14.1/0.3	25.6/0.4	${\textbf {11.4/0.9}}$	466.5/0.9
100	10	${\textbf {29.4/0.7}}$	39.5/1.1	76.9/2.6	4531.1/1.7
105	11	${\textbf {74.6/1.6}}$	91.9/2.1	339.6/3.6	$+$
110	11	${\textbf {102.9/1.6}}$	130.2/2.5	437.3/5.3	$+$
115	12	${\textbf {190.2/2.9}}$	256.0/3.6	809.9/6.6	$+$
120	13	${\textbf {1151.1/12.3}}$	1362.8/17.6	$+$	$+$

As shown in Table 2, the time consumption of Fast-LCS and Quick-DP increases dramatically as the length of DNA sequences increases. However the time consumption of Top-MLCS and mini-MLCS increases considerably more slowly. Additionally, Quick-DP runs faster than Top-MLCS and mini-MLCS when the sequence length is less than 95. Quick-DP outperforms Top-MLCS and mini-MLCS in these circumstances due to the short time required for non-dominated sorting.

**Table 3:** The average results for benchmark BL, length fixed to 100

Number of sequences	$|\Sigma |$	A*			Top-MLCS			mini-MLCS		
		${\overline{R}}$	${\overline{t}}$	$\#$opt	${\overline{R}}$	${\overline{t}}$	$\#$opt	${\overline{R}}$	${\overline{t}}$	$\#$opt
10	4	20.5	428.33	6	0.0	–	0	20.5	**393.76**	6
	12	12.7	1.73	10	12.7	5.2	10	12.7	**0.78**	10
	20	7.9	0.08	10	7.9	0.28	10	7.9	**0.07**	10
50	4	0.0	–	0	0.0	–	0	20.1	**374.20**	7
	12	6.9	0.17	10	6.9	0.46	10	6.9	**0.11**	10
	20	3.0	0.06	10	3.0	0.08	10	3.0	**0.05**	10
100	4	0.0	–	0	0.0	–	0	19.3	**311.36**	6
	12	5.2	0.08	10	5.2	0.23	10	5.2	**0.05**	10
	20	2.1	0.07	10	2.1	0.08	10	2.1	**0.04**	10
150	4	0.0	–	0	0.0	–	0	18.8	**280.54**	9
	12	4.7	0.07	10	4.7	0.16	10	4.7	**0.05**	10
	20	1.9	0.08	10	1.9	0.08	10	1.9	**0.03**	10
200	4	0.0	–	0	0.0	–	0	18.0	**180.24**	8
	12	4.1	0.07	10	4.1	0.18	10	4.1	**0.06**	10
	20	1.1	0.06	10	1.1	0.11	10	1.1	**0.01**	10

The bold values represent the shortest average running time of all the algorithms in the table on the dataset

Table 3 lists average solution lengths ${\overline{R}}$, average times ${\overline{t}}$ in seconds until proven optimality has been reached, and the number of instances that could be solved to optimality $\#$opt (out of ten per line) for three approaches. It can be seen that mini-MLCS is more efficient than Top-MLCS and A* in processing small instance sets because it filters most of the match points, it will not cause memory overflow. However, none of the instances with $|\Sigma |$ = 4 and *d* $\ge$ 50 could be solved to optimality by the A* and Top-MLCS due to the memory limit. As shown in Table 4, there is one parameter *t* in the proposed algorithm for calculating the lower bound *Lower*(*R*) of the length of MLCS. To assess the estimate method’s robustness, we examine the influence of *Lower*(*R*) by altering the values of *t* throughout the experiments. The more exact the predicted lower limit *Lower*(*R*) is, the more unnecessary match points can be found and removed from the DAG. The experiments on problems include 20000 sequences of varying lengths between 90 and 120. As can be observed from the experimental results, for each test problem with a constant length, the values of *Lower*(*R*) are very little affected by changes in the values of *t*. This indicates that the effect of *t* on *Lower*(*R*) is negligible and that the *Lower*(*R*) estimate approach is robust.

**Table 4:** The *Lower*(*R*)/run time(s) generated by different change in *t*

Length of sequences	*R*	*Lower*(*R*)				
		10	20	50	100	150
90	8	8/0.06	8/1.63	8/2.73	8/4.13	8/5.79
95	9	8/1.28	8/1.88	9/2.60	9/4.88	9/6.68
100	10	10/1.15	10/2.28	10/4.99	10/8.77	10/10.81
105	11	11/0.77	11/2.50	11/3.47	11/10.60	11/9.37
110	11	10/1.68	11/2.74	11/4.35	11/10.19	11/13.03
115	12	10/1.55	11/2.85	11/6.75	12/11.29	12/15.77
120	13	12/1.07	12/2.53	12/4.21	13/6.38	13/10.21

## Conclusion

This paper proposed a unique branch elimination strategy(mini-MLCS) by identifying the useless match points for effectively and efficiently tackling large-scale MLCS problems. Which quickly identifies unnecessary match points during the construction of the DAG, reducing the time spent in non-dominated sorting each level of the previous DAG. The experimental results show that it outperforms current state-of-the-art algorithms Fast-LCS, Quick-DP and Top-MLCS, and is capable of addressing large-scale MLCS problems. The approach takes much less time and space than Fast-LCS, Quick-DP, and Top-MLCS, particularly for large-scale MLCS problems.

## Data Availability

This program code can be available at https://github.com/BioLab310/mini_MLCS. Biological sequences from NCBI (http://www.ncbi.nlm.nih.gov/nuccore/110645304?report =fasta) are selected as the test sets.
